# Scientific Items

**Published:** 1888-04

**Authors:** 


### SCIENTIFIC ITEMS.

**Warning Apparitions.**—As to the question of fact involved in these stories, he says ; "It appears to me that the evidence regarding the communication of impressions from mind to mind over great distances, in such sort that apparitions of distant persons dying or suffering seem to be seen by their friends or relatives, is too strong to be rejected by any conscientious student of facts." He believes that science is no more justified in rejecting this evidence merely because no explanation is available than astronomers would be justified in rejecting the observed facts that bodies influence other bodies from a distance, merely because, as Newton himself admitted, no one can explain how matter can act where it is not. Some communication, he says, there must be between sun and planet, between planet and satellite, and beyond each solar system between sun and sun and between galaxy and galaxy ; but no one has yet shown what that communication may be. In like manner, even the most cautious student of science may well believe that there may be some means of communication, under special conditions, between mind and mind at a distance, though no one may be able to explain how such communication is brought about.—*Medical and Surgical Reporter.*

**Savagery in Boyhood.**—Every thing, we suppose, must be considered hereditary in the present age ; even the tendency to wear cocked hats, or to throw cabbage-heads on hallow eve. At any rate, the *Popular Science Monthly* for October brings this doctrine to bear upon the phonomena of savagery in boyhood, as noticed in *Science* of October 7. The author explains that cruelty in children is the transmitted habit of ancestral savages, and observes that "the emotion of pity appeared late in the history of the race." In the same connection we may mention the intense interest which children take in narratives of warfare : torturing animals is a less general incident. But the callousness of children in contemplating the horrors of war and its consequences has always been an interesting fact to us. However, is no other analysis of this possible than the supposition that our savage forefathers were cruel?—*Science.*

**A Dental Matrix** has been patented by Mr. John H. Reed, of Lancaster, Wis. It consists of a hard metal yoke, with a screw, and a band of softer metal, to be attached to the yoke by tongues passing through apertures in the yoke, making a device for supporting the filling while being inserted in a cavity in a tooth, supplying the place of one or more of the natural walls of the tooth cavity.—*Scientific American.*

**An** Australian mineral called maldonite has been found to consist of an alloy of gold and bismuth, containing sixty-four per cent. of the former metal.

**The Influence of Forest upon Rainfall and Climate.**—Aside from the beneficial influence of forests in the retention and saving of the water which falls, may it not be that there is an effect of the forest upon climatic extremes of heat and cold? This is well shown, I think, by the experience of western Michigan. During the early years of the settlement of the country, before the forests were destroyed, all the delicate fruits of temperate climates were successfully grown.

Since the forests are nearly gone, the tender varities of peaches can no longer be raised, except in a few favored localities, on account of extreme winter cold; and the heat of our summers has been of late years as extreme as the cold of our winters.—*H. D. Post in Science.*

**The** *British Medical Journal* reports a case of leprosy which is beleived to have been contracted through vaccination. A physician living in the tropics vaccinated his own son from virus obtained from a native child in whose family leprosy existed. At the time the virus was taken, the child gave no evidence of being affected with the disease, although subsequently it manifested itself in him. A third child was vaccinated by the physician with virus taken from his own son. Subsequently the son developed leprosy in a mild form; but the child who was vaccinated with virus taken from him had the disease in a most severe form, and died from it. The physician's son is now attending school in England, eminent physicians having given the opinion that there is no danger that the othes will contract the disease.

**A New Gas.**—*Public Opinion*, February 11, 1888, states that a new gas has been discovered in Germanny by Dr. Theodore Curtis, who has succeeded in preparing the long-sought hydride of nitrogen, amidogen, diamide, or hydrazine, as it is variously called. This remarkable body, which has hitherto baffled all attempts at isolation, is now shown to be a gas perfectly stable up to a very high temperature, of peculiar odor—differing from that of ammonia—exceedingly soluble in water, and of basic properties. In composition it is nearly identical with ammonia, both being compounds of nitrogen and hydrogen.—*Medical and Surgical Reporter.*

**A Laryngoscope** has been patented by Mr. Josef Leiter, of Vienna, Austria-Hungary. It has an electric lamp within a cylindrical casing, and a reflector for throwing the rays into the cavity to be examined and in line with the eye of the operator, with other novel features, making an improved surgical instrument for viewing interior parts of human and animal bodies.—*Scientific American.*

**Wrought-iron** expands and contracts with a force of about two hundred pounds to the square inch for each degree Fahenheit.

